# Parental Misperceptions of Their Offspring’s Weight and Their Strategies for Child’s Eating Behavior: A Narrative Review of the Recent Evidence

**DOI:** 10.3390/children9101565

**Published:** 2022-10-16

**Authors:** Ioannis Gketsios, Alexandra Foscolou, Tonia Vassilakou, Demosthenes B. Panagiotakos, Rena I. Kosti

**Affiliations:** 1 Department of Nutrition and Dietetics, School of Physical Education, Sports and Dietetics, University of Thessaly, 42132 Trikala, Greece; 2 Department of Nutrition and Dietetics, School of Health Sciences and Education, Harokopio University of Athens, 17671 Athens, Greece; 3 Department of Public Health Policy, School of Public Health, University of West Attica, 11521 Athens, Greece; 4 Faculty of Health, University of Canberra, Canberra 2617, Australia

**Keywords:** parents, children, weight, perceptions, misperceptions, eating behaviors, strategies, interventions

## Abstract

The aim of the present review was to explore the effect of parental misperceptions of their offspring’s weight status during childhood and early adolescence on weight control strategies and children’s eating behavior. Literature searching was limited to the PubMed database and to the English language from January 2000 to August 2022. Eligible studies had clearly associated parental misperception of offspring’s weight with child eating habits or weight management and eating strategies in childhood to early adolescence. Sixteen studies (14 cross-sectional, 1 longitudinal and 1 with cross-sectional and longitudinal analyses) were included in the analysis. Weight loss attempts and child’s eating behavior were the main outcomes. Sixteen studies found significant associations. Parental misperceptions of their offspring’s weight status do influence their child’s weight and eating behavior, especially in overweight children. Parents tend to follow potentially harmful methods when they overestimate their children’s weight (food restriction) and when they underestimate their children’s weight (pressure to eat). However, additional longitudinal studies are needed to better understand the impact of parental weight status perception on health behaviors and children’s weight gain over time. The potential need for preventive intervention studies is warranted.

## 1. Introduction

Obesity is a major public health problem. Obesity stemming from childhood often projects into adulthood with well-known devastating health consequences [1,2]. Prevention of obesity in childhood and effective treatment of overweight children are crucial steps towards the restriction of the obesity epidemic. Actually, a lot of clinicians have presented the significance of pediatric weight clinics using a holistic method for treatment [3]. However, during childhood and early adolescence, parents can still effectively influence their children’s eating behavior and lifestyle. Parental attitudes towards their child’s weight status, as well as parental obesity, create a “weight culture” in the family not irrelevant to their social environment and may well affect children’s eating behavior and general lifestyle until early adolescence [4,5,6].

Parents often misperceive their offspring’s weight status, especially in early-middle childhood [7,8]. It is estimated that half of parents incorrectly classify their child’s weight and misclassification can reach up to 90% [7,9,10]. This fact may interfere with weight control and tackling obesity campaigns since they tend to underestimate the weight of their apparently overweight or obese children. Nevertheless, evidence on the association of weight misperception with lifestyle and feeding practices is scarce and inconsistent [11]; parental recognition of their child being overweight is not necessarily translated into healthy changes in weight-related parenting behaviors or child behaviors [12,13], and further research should be directed toward better understanding the impact of weight status perception on health behaviors and weight gain over time [14].

The 5–7-year-age period is regarded as the beginning of the ‘‘age of reason’’ [15]. Children are assigned roles, and they slightly start to socialize, opening gradually to various environmental influences. Parents’ misperception of children’s weight status reflects their concern and may influence parental feeding practices, children’s dietary quality and weight status in various ways [16,17,18,19]. Despite abundant studies about parental weight misperceptions, the well-recognized necessity for overweight prevention strategies in school-aged children and the importance of parental influences in childhood, little is known about how the parental misperception of their offspring’s weight may influence the weight management actions and eating behaviors of their children. Thus, the aim of the present study was to review the recent studies exploring the effect of parental misperceptions of their offspring’s weight status during childhood and early adolescence on weight control strategies and children’s eating behavior.

## 2. Literature Search

**Figure 1:**
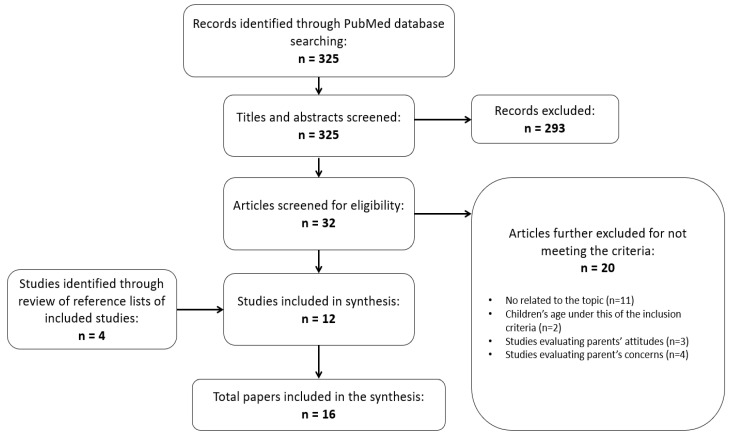
Flowchart of the selection process of the literature-search.

A computer-assisted literature review was performed using the PubMed database. The search was narrowed to articles between 1 January 2000 and 31 August 2022. In this narrative review, used search strings were: “parent*” AND (“perceptions” OR “misperceptions”) AND (“weight” OR “weight status”) AND “child*” AND (“diet*” OR “behavior*” OR “feeding” OR “practices” OR “eating” OR “strategies”). Restricted to humans, children and young adolescents (i.e., ages that parents play an essential role in influencing how adolescents interact with the factors that shape their development [20]), as well as the English language, 325 hits were obtained from the PubMed search. The flow-chart of the selection process of the literature search is shown in Figure 1. By removing articles not relevant (i.e., articles excluded upon screening the title and/or the abstract, articles where no association between parental misperception and diet/weight modification was considered, reviews, and articles not meeting the inclusion criteria), the number of articles retained for this review was 18. Two reviewers (IG, AF) independently selected the articles, and any discrepancies between them were solved by consensus or by consulting with a third researcher (RK).

### 2.1. Eligibility Criteria

Eligible studies had to meet the following inclusion criteria: (i) clearly associated parental misperception of offspring’s weight with child eating habits or weight management and eating strategies, (ii) children or adolescents between 6 and 15 years of age, with no child being over 15 years of age, i.e., entering from early to middle childhood and extended up to young teenage, leaving out middle and late adolescence period. We included studies with children and early adolescents since, during childhood and early adolescence, parents can still effectively influence their children’s eating behavior and lifestyle.

### 2.2. Data Extraction

From each study, the following relevant data were extracted: first author, year of publication, country, study design type, sample size, age of children, parental perception assessment, diet assessment, BMI reference norms and the key results/conclusions of each study.

## 3. Study’s Findings

In this study, we reviewed former relevant published studies to investigate the effect of parental misperceptions of their offspring’s weight status during childhood and early adolescence on weight control strategies and children’s eating behavior. This narrative review showed a total of sixteen studies related to the aforementioned association (Table 1).

### 3.1. General Characteristics of the Studies

Table 1 shows the general characteristics of the 16 studies included in this review (14 cross-sectional studies, one study that retrospectively analyzed data from two longitudinal studies and one with cross-sectional and longitudinal analyses). The studies took place in Europe [*N* = 5, i.e., 8 European countries (*n* = 1) including Belgium, Greece, Hungary, Netherlands, Norway, Slovenia, Spain, Switzerland, and the UK (*n* = 4)], in Asia [*N* = 3, i.e., China (*n* = 3)], in America [*N* = 6, i.e., the USA (*n* = 6)], in Australia (*n* = 1) and from the network (*n* = 1). The sample size varied from 49 individuals [21] to 47,417 [19], while age range varied from 3–15 years old [29] to 6–18 years old [32]. However, since the aim of the study was to investigate the effect of parental misperceptions of their offspring’s weight status during childhood and early adolescence on weight control strategies and children’s eating behavior, we are presenting only the results for ages between 6 and 15, i.e., ages that parents have an essential role in influencing their children. The following BMI reference norms were mainly used to compare actual weight with perceived weight: IOTF [11,16,30,32], National charts/CDC norms [21,25,26,27,32,33].

### 3.2. Parental Weight Misperceptions Assessment

For the assessment of parental perceptions, the following methods were applied: interview with quantitative techniques [30], interview with CAPI questions [25]. In all other studies presented in Table 1, one question with three to six responses and different phrasing was used to assess children’s weight status. For example, in the Tarasenko et al. (2014) study, parents were asked if their child was “fat or overweight,” “too thin” or “about the right weight.”, while in the study of Webber et al. (2010) mothers were asked if their child was “very underweight”, “underweight”, “normal”, “overweight” or “very overweight”.

However, different questions were used to assess parental perception of their child’s weight. For example, in the study of Almoosawi et al. (2016), parents’ perception of their child’s weight status was assessed using the question ‘How would you describe your child’s weight at the moment?, while in the study of Robinson & Sutin [32], the respective question was ““Which of these best describes your child?” The response options were “underweight,” “normal weight,” “somewhat overweight” and “very overweight”.

### 3.3. Evaluation of Dietary Habits

Regarding the evaluation of dietary habits, the Child Feeding Questionnaire was the most used tool [21,22,24,27,33]. Diet was assessed using the FAST (Food Assessment in Schools Tool) food diary method in the study of Almoosawi et al. (2016). Meanwhile, in some cases, children were directly asked about their eating behavior, e.g., Altenburg et al. (2016), while in others e.g., Vangeepuram et al. (2016), only parents were asked. Weight loss attempts were reported by the children themselves. Of note, data on slimming behavior were collected from both parents and children in the study of Altenburg et al. (2016). No standardized questionnaires were used to collect parents’ data on children’s nutrition in the studies of Crawford et al. (2005) and Vangeepuram et al. (2016).

### 3.4. Prevalence of Parental Weight Misperception

Percentages of parental weight misperception (parents perceived their child as being of ‘normal’ weight when they were actually ‘overweight’ or ‘obese’) ranged from 17% [29] to the highest 80% [32]. It should be noted that there is a large discrepancy between studies regarding how parents perceive their children’s weight. As presented in Table 1, the percentages of parental misperception do not follow a specific pattern.

### 3.5. Association between Parental Weight Misperception and Eating Habits/Weight Loss Attempts

Four out of the sixteen studies included in the present review explored parental weight misperceptions in association with weight loss attempts of the children (this was indirectly examined in the study of Altenburg et al. (2016) under the term “slimming behavior”). In the other twelve studies, parental misperceptions were explored in association with child eating behavior and parental attitudes towards healthy eating.

More specifically, it was revealed that children whose parents perceived them as overweight were more likely to attempt losing weight [32], and if this perception was made by at least two persons, a child was twice as likely to attempt weight loss [26]. However, one study found that parental misperceptions were not significantly associated with attempted weight loss of children/adolescents [25]. Nevertheless, even in this study, in the overweight group, Hispanic blacks were more likely to report attempts than non-Hispanic whites, a finding implying some effect of misperceptions on certain cultural subgroups [25].

Regarding healthy eating behavior and parental perceptions, it was revealed that children whose parents misperceived their weight status did not follow the optimal dietary pattern [29] and that when parents underestimated their child’s weight, those children usually watch television during their meals [29] and spend fewer hours a day walking [28]. On the other hand, children whose parents accurately classify their children’s body weight have more healthy habits, e.g., daily breakfast, more exercise, less soft drink consumption and less screen time [19], and generally their parents try encouraging methods to ameliorate their children’s physical activity level [24] and eating habits [10].

Finally, two studies revealed that parents who perceived their children as overweight or obese use restriction eating methods, e.g., always watching the quantity and the quality of children’s food consumption or hiding specific foods from their children [27,33]; while mothers who perceive their child as underweight use more pressuring strategies in order for them to eat, e.g., making sure that their child follows specific food rules or feeding their child even if the child is not hungry [21,22,23,27].

### 3.6. Perceptions and Strategies Based on Sex, Age Group and Study Design

Four out of the sixteen studies evaluated separately maternal and paternal perceptions and strategies. It was found that mothers were more likely to use pressuring methods to eat if they believe that their child’s weight is below the normal [22] and more likely to encourage their child to engage in physical activities and change their child’s diet [31]. Moreover, mothers were more likely to correctly identify their child’s weight status [23]. Accordingly, fathers of boys with an average BMI more often use pressuring and monitoring methods compared to fathers of boys with high BMI [21].

It is worth noticing that parents behave differently based on the sex of their child. For example, parents are more likely to wrongly identify a boy’s weight than that of a girl’s [19,23], while they use more restrictive practices if they classify their boy’s weight as above normal compared to parents who classify their daughters as overweight [33].

Accordingly, parents behave differently based on the age of their children. For example, parents were more likely to underestimate children’s weight status than that of adolescents [19,27], even though one study found the opposite [32]. Furthermore, a greater proportion of parents of 10–12-year-old children reported using preventive strategies for weight gain compared to parents of 5–6-year-old children, while a greater proportion of parents of 5–6-year-old girls reported weight gain prevention than parents of same-age boys [16].

Finally, in the study that used cross-sectional and longitudinal analyses, it was revealed that more parents misclassify the weight status of their adolescent sons than of their daughters, while 50% vs. 43.9% of the overweight boys and girls who had not been encouraged to diet, respectively, remained overweight compared to the 74.1% and 66.1% of boys and girls who had been encouraged to diet [10].

## 4. Discussion and Concluding Remarks

The present narrative review, summarizing the effect of parental misperceptions of their offspring’s weight status during childhood and early adolescence on weight control strategies and children’s eating behavior, showed that parental weight misperception may influence children’s weight and the strategy each parent will follow to improve eating behaviors, especially in overweight children. It seems that parents who accurately or not perceived that their child was overweight reported restrictive feeding practices and encouraging methods to modify their children’s eating habits. At the same time, parents who underestimated their children’s weight were more likely to follow harmful strategies for their children. Moreover, it seems that European parents were more inconsistent regarding their perception about the actual body status of their children compared to Asian, American or Australian parents. Based on the above-mentioned results, these can be characterized as particularly important for the future course of life and health of children, who will constitute the human resources of a healthy society.

The role of the parent Is one of the most important and, unfortunately, there is no instruction manual. No matter how hard parents try, they will also make mistakes raising their child, as their roles are multiple [34]. It is true that the family may influence the dietary intake and behavior of the child in various ways, interacting with the environment to which it belongs. Parents often express their willingness to improve their child’s eating habits, trying to increase the consumption of foods that are considered healthy choices and, at the same time, weaken the consumption of those that should be eaten less often. However, the practices they usually use, namely restriction, reward or pressure to consume, can lead to the opposite of the originally intended results [35].

Moreover, all the before-mentioned results could possibly be explained by the fact that parents’ perceptions of their child’s weight are also shaped by cultural, socioeconomic and psychological factors. Factors such as the child’s gender and age, ethnicity, parent’s weight, increased daily caloric intake of the parent, low income and low educational level affect the ability of parents to correctly estimate their child’s weight [36]. It seems that the level of education of the parents as well as the educational level of the mother positively affect the consumption of healthy foods. Besides, children whose mothers have a low education tend to follow a diet that contains unhealthy options. On the contrary, as the educational level of the parents increases, the quality of the children’s diet improves [37]. It is no coincidence that socioeconomic status and obesity are directly linked and vary from country to country [38]. Accordingly, one possible explanation for parental weight misjudgment is the idealization or desirability theory, according to which individuals perceive things more favorably than they actually are. So, parents may have difficulty recognizing normal weight because they tend to idealize their child and think that they are not fat, but normal [39].

Inaccurate perceptions begin very early, in infancy. Pediatricians, nutritionists, and health scientists should help parents improve their understanding of the actual body weight of their children. The parental perceptions of a child’s development and correct eating behavior are the factors that will determine the future weight status of a child. Also, they are the ones that will be a key factor for the family, leading them to modify their eating behavior and change their general way of life [40]. Actually, it is crucial for parents to accurately identify their child’s weight status to prevent obesity [41].

To the best of our knowledge, this narrative review paper is one of the first of its kind to summarize the effect of parental misperceptions of their offspring’s weight status on weight control strategies and children’s eating behavior. However, this review has some limitations that need to be addressed. The observational nature and the epidemiological evidence presented from observational studies are more prone to bias and confounding and cannot be used to demonstrate causality. Besides, the heterogeneity of research tools applied to evaluate parental perceptions may be considered as another limitation. Regarding the review process, it was limited to the PubMed database and to the English language. However, as data paucity on the interplay between parental weight misperception in early-middle childhood and child’s healthy behavior is well recognized, it seems unlikely that any further publication would substantially alter our findings.

In conclusion, the aggregated evidence corroborates that parental weight misperceptions do influence their child’s weight and eating behavior, especially in overweight children. Meanwhile, parents seem to follow restrictive eating methods when they believe, accurately or not, that their child is overweight or obese, while parents who underestimate their children’s weight are more likely to pressure them to eat, i.e., two strategies potentially harmful for children. However, the association between parental misperceptions and the weight control strategies implemented needs further investigation. Systematic observations and prospective studies may shed further light on the issue and allow the appropriate community interventions. Further studying the effect of parental misperceptions on weight control strategies may provide valuable information for educating families and promoting healthier lifestyles in early adolescence with apparent benefit in adulthood.

## Figures and Tables

**Table 1:** Characteristics of the 16 retained studies which assess the relationship between parental weight misperception and child’s eating habits and weight loss attempts.

Source (Chronological Order)	Study Design	Subjects (Age, Country)	Parental Perception Assessment	Dietary Habits Assessment	BMI Reference Norms	Perception Results	Strategies	Key Results/Conclusions
1. Brann & Skinner, 2005 [21]	Cross-sectional	8–10 years old (*n* = 49/USA)	n/a	Child Feeding Questionnaire	Centers for Disease Control charts	High BMI vs. Average BMI boys Both parents saw them as more overweight	Average BMI boys Both parents: Controlling feeding practices Mothers: Pressure to eat Fathers: Pressure to eat & monitoring More often than mothers/fathers of boys with a high BMI	Boys with average BMI have parents using more controlling child-feeding practices compared to boys of high BMI.
2. Crawford et al., 2006 [16]	Cross-sectional	5–6 years old, 10–12 years old (*n* = 1205/Australia)	Classification using a 5-point scale: “Markedly underweight”, “Underweight”, “Average”, “Overweight”, “Markedly overweight”	n/a	International Obesity Task Force, 2000	5–6 years old children 3% of parents perceived their children as overweight/obese (23% of the children were actually overweight/obese) 10–12 years old children 14% of parents perceived their children as overweight/obese (29% of the children were actually overweight/obese)	5–6 years old girls vs. boys: More often weight gain prevention strategies (39% vs. 24%, *p* < 0.005) 10–12 years old vs. 5–6 years old More parents reported using preventive strategies for weight gain (31% vs. 43%, *p* < 0.001)	Overall, preventive strategies were not associated with current child-weight perception.
3. Neumark-Sztainer et al., 2008 [10]	Cross-sectional and Longitudinal	Mean age 14.4 ± 1.7 years old (*n* = 314 included in cross-sectional analyses and *n* = 170 in longitudinal analyses/USA)	Classification using a 6-point scale: “Very underweight”, “Somewhat underweight”, “About right”, “Somewhat overweight”, “Very overweight”, “Don’t know”	Questionnaire on lifestyle behavior and eating habits	n/a	48.5% of obese parents misperceived the overweight body of their children 55.2% of non-obese parents misperceived the overweight body of their children 45.9% and 60% of parents misclassified their daughters and sons, respectively, as being of normal weight	50% and 43.9% of boys and girls, respectively, who had not been encouraged to diet remained overweight vs. 74% and 66.1% who had been encouraged	Overall, parents who recognized that their children were overweight were more likely to encourage them to diet.
4. Webber et al., 2010 [22]	Cross sectional	7–9 years old (*n* = 213/UK)	Classification using a 5-point scale: “very underweight”, “underweight”, “normal”, “overweight”, “very overweight”	Child Feeding Questionnaire	1990 British reference data	59% vs. 56% maternal misperception of their underweight and overweight/obese children, respectively	Pressure to eat was the most common strategy for mothers who perceived their children as being underweight.	Maternal practices depend on their perception of their child’s weight, especially when mothers perceive their children as being underweight.
5. Wen & Hui, 2011 [23]	Cross-sectional	10–15 years old (*n* = 2143 adolescents and *n* = 1869 parents/China)	Classification using a 5-point scale: “Very underweight”, “Slightly Underweight”, “Normal”, “Slightly Overweight”, “Very overweight”	Lifestyle and nutritional questions	International growth standards of WHO	44% of parental misperception Parents are more likely wrongly identify their son’s weight than that of their daughter’s OR: 1.61, 95%CI: 1.29–2.01	Parents who correctly classified their children: “monitoring diet and physical activity” “positive reinforcement” vs. “pressure to eat” for parents who misperceived their child’s weight	Parents with incorrect perception of their children’s weights were more likely to select the feeding strategy of ‘pressure to eat’ compared with parents with correct perception.
6. Polfuss & Frenn, 2012 [24]	Cross-sectional	9–15 years old (*n* = 176 child parent dyads/USA)	Classification using a 5-point scale: “Markedly underweight”, “Underweight”, “Average”, “Overweight”, “Markedly overweight”	Child Feeding Questionnaire	BMI z scores standardized for age and gender according to the national norms from the USDA/ARS Children’s Nutrition Research Center	Parental perception of their child’s weight was significantly correlated with BMI z scores (*p* < 0.001).	Psychological control and pressure to eat were significantly associated with the child’s weight status.	Overall, parents who accurately perceived their child as being overweight used controlling behaviors.
7. Chen et al., 2014 [25]	Cross-sectional	8–15 years old (*n* = 2613/USA)	Computer-Assisted Personal Interview (CAPI) questions. Classification: “overweight”, “underweight”, “about the right weight”	Weight loss attempts	Centers for Disease Control charts	6.3% of parents perceived their child’s weight as underweight, 74.4% as about right, 19.3% as overweight	Only correct personal weight perception was positively associated with self-reported attempted weight loss for overweight or obese children.	Parental misperceptions were not significantly associated with attempted weight loss of children/adolescents.
8. Tarasenko et al., 2014 [26]	Cross-sectional	8–15 years old (*n* = 4691/UK)	Early childhood questionnaire Classification: “overweight”, “underweight”, “about the right weight”	Weight loss	US growth charts	Probability that an overweight child is perceived as such by their guardians 0.25 (95%CI: 0.20–0.30) vs. an obese child: 0.41 (95% CI: 0.38–0.45)	Overweight children 63% more likely (95% CI: 1.30–2.04) to attempt weight loss when only one person accurately recognizes them as such vs. overweight children misclassified by at least one person (*p* = 0.001)	Overweight children perceived as such by two people [i.e., themselves and their guardians or health care professionals (HCPs), or both their guardians and HCPs] were twice as likely to attempt weight loss compared to their counterparts perceived as such by nobody.
9. Lydecker & Grilo, 2016 [27]	Cross-sectional	5–15 years old (*n* = 1007/Mechanical Turk website -multiracial)	Classification using a 4-point scale: “Underweight”, “Healthy weight”, “Overweight”, “Obesity”	- Child Feeding Questionnaire - Eating Disorder Examination Questionnaire - Fat Talk Questionnaire	Age-, sex- specific growth curves	47.7% misclassified their underweight child 24.3% misclassified their healthy weight child 73.1% misclassified their overweight child	Perceived child weight-status was related to “restriction” (*p* < 0.001), “pressure to eat” (*p* < 0.001) and “monitoring” (*p* = 0.003).	Parents restricted the eating of children with perceived overweight and obesity more than perceived underweight and healthy weight children. Besides, they pressured underweight children to eat more than those with healthy weight, overweight, and obesity, while they monitored obese children more than underweight ones.
10. Vangeepuram et al., 2016 [28]	Cross-sectional	3–15 years old (*n* = 116, USA)	Classification using a 4-point scale: “Underweight”, “About the right weight”, “Somewhat overweight”, “Very overweight”	Questions on lifestyle behavior, no standardized questionnaire reported	n/a	12% and 81% of parents underestimated their children’s healthy and overweight/obese weight, respectively	Perceiving the child’s weight as normal was associated with more hours of daily walking (*p* = 0.08).	Perceptions of a child’s weight may be related with parental perception of child health and physical activity status.
11. Almoosawi et al., 2016 [29]	Cross-sectional	6–8 years old (*n* = 361/UK)	Interview (quantitative techniques) Classification: “very underweight”, “underweight”, “normal” “overweight” “very overweight”	Food Assessment in Schools Tool	International Obesity Task Force	17% of parents misperceived their child as being of normal weight 0% of parents overestimated their child’s weight	More children with misperceived weight consumed reduced sugar carbonated drinks (*p* = 0.005).	Parents who misperceived their child’s weight had children with a lower ‘healthy’ dietary pattern score compared to children whose parents correctly perceived their weight
12. Altenburg et al., 2017 [30]	Cross-sectional	10–12 years old, (*n* = 7708/Belgium, Greece, Hungary, Netherlands, Norway, Slovenia, Spain, Switzerland—ENERGY project)	Classification using a 5-point Likert scale: “My child’s weight is OK”, “My child’s weight is a bit too much”, “My child’s weight is way too much”, “My child’s weight is a bit too little”, “My child’s weight is way too little”,	Standardized battery/weight loss-slimming behavior	Slimming and energy-balance related behaviors	22% of parents perceived their children’s weight as “a bit or way too much” when 23% of children were actually overweight or obese	Children whose parents perceive them as overweight reported less physical activity (β = −3.4).	Children whose parents perceive them as overweight reported less physical activity.
13. Min et al., 2017 [31]	Cross-sectional	6–18 years old (*n* = 816/China)	Classification using a 3-point scale: “Underweight”, “Normal weight”, “Overweight”	24 h recall and same-day interview	Obesity Task Force BMI cut-offs	36.2% of mothers misperceived their child’s weight, 22.2% under-perceived and 14% over-perceived their child’s weight	Mothers who perceived their child’s weight as overweight -encourage the child to exercise more (Odds Ratio = 1.8, 95%CI: 1.0–3.3) -encourage the child to change weight with diet (Odds Ratio = 4.3, 95%CI: 2.3–7.8)	Mothers who perceive their child as overweight are more likely to encourage their child to exercise and modify their diet for weight management.
14. Robinson & Sutin, 2017 [32]	Longitudinal *	Study 1 4–5 years old→ 14–15 years old (*n* = 2823/UK) Study 2 9 years old→ 13 years old (*n* = 5886/UK)	Study 1 Classification using a 4-point scale: “Underweight”, “Normal weight”, “Somewhat overweight”, “Very overweight” Study 2 Classification using a 7-point scale: “Very underweight”, “Moderately underweight”, “Slightly underweight”, “About the right weight”, “Somewhat overweight” “Moderately overweight”, “Very overweight”	Weight loss attempts	Centers for Disease Control charts	Baseline 4–5 years old 19% of actually overweight or obese children were identified as such by their parents 9 years old 44% of actually overweight or obese children were identified as such by their parents Overall 80% of overweight/obese children were identified as normal by their parents	Children perceived as overweight Point estimate: 0.155 ± SE: 045 (*p* < 0.01) for child weight-loss attempts	Children whose parents perceive them to be overweight were more likely to actively try to lose weight.
15. Zhang et al., 2018 [19]	Cross-sectional	6–17 years old (*n* = 47,417/China)	Classification using a 3-point scale: “thin”, “about the right weight”, “fat”	Standardized behavioral and nutritional questionnaires	BMI Z-score using WHO standards	30.5% of parental underestimation and 8.7% of parental overestimation of their child’s weight The weight of girls was less likely to be underestimated than that of boys (25.1% vs. 35.8%, *p* < 0.001)	Parents use healthy strategies towards their child’s dietary habits (breakfast, exercise, screen time and soft drinks restriction) if they classify them as of normal weight.	Parents who perceived that their child has normal weight use healthy strategies towards child’s dietary habits
16. Loth et al., 2021 [33]	Cross-sectional	5–7 years old (*n* = 150/USA)	Classification using a 6-point scale: “Very underweight”, “Somewhat underweight”, “About right”, “Somewhat overweight”, “Very overweight”, “Don’t know”	Child Feeding Questionnaire (CFQ)	Centers of Disease Control calculator	n/a	Parents who perceived their child to be somewhat/very overweight reported more restrictive feeding practices compared to those who perceived their child to be very or somewhat underweight or about right weight.	Parental perception of their child’s weight was associated with parental report of restrictive feeding practices

* Retrospectively analyzed data from two longitudinal studies from different countries.

## Data Availability

Not applicable.
